# Refraining from pre-hospital advanced airway management: a prospective observational study of critical decision making in an anaesthesiologist-staffed pre-hospital critical care service

**DOI:** 10.1186/1757-7241-21-75

**Published:** 2013-10-25

**Authors:** Leif Rognås, Troels Martin Hansen, Hans Kirkegaard, Else Tønnesen

**Affiliations:** 1 Department of research and development, Norwegian Air Ambulance Foundation, P.O. Pox 94, 1441 Drøbak, Norway; 2 Department of Anaesthesiology, Pre-hospital Critical Care Team, Viborg Regional Hospital, Heibergs Allé 4, 8800 Viborg, Denmark; 3 Pre-hospital Critical Care Team, Aarhus University Hospital, Trindsøvej 4-10, 8100 Aarhus C, Denmark; 4 Department of Pre-hospital Medical Services, Central Denmark Region, Oluf Palmes Allé 34, 8200 Aarhus N, Denmark; 5 Centre for Emergency Medicine Research, Aarhus University Hospital, Trøjborgvej 72-74, Building 30, 8200 Aarhus N, Denmark; 6 Department of Anaesthesiology, Aarhus University Hospital, Nørrebrogade 44, 8000 Aarhus, Denmark

**Keywords:** Pre-hospital, Out-of-hospital, Prehospital emergency care (MeSH), Emergency medical services (MeSH), Helicopter emergency medical service, Critical care (MeSH), Airway management (MeSH), Endotracheal intubation (MeSH), Patient safety, Critical decision making (MeSH)

## Abstract

**Introduction:**

We report prospectively recorded observational data from consecutive cases in which the attending pre-hospital critical care anaesthesiologist considered performing pre-hospital advanced airway management but decided to withhold such interventions.

**Materials and methods:**

Anaesthesiologists from eight pre-hospital critical care teams in the Central Denmark Region (a mixed rural and urban region with 1.27 million inhabitants) registered data from February 1^st^ 2011 to October 31^st^ 2012. Included were patients of all ages for whom pre-hospital advanced airway management were considered but not performed. The main objectives were to investigate (1) the pre-hospital critical care anaesthesiologists’ reasons for considering performing pre-hospital advanced airway management in this group of patients (2) the pre-hospital critical care anaesthesiologists’ reasons for not performing pre-hospital advanced airway management (3) the methods used to treat these patients (4) the incidence of complications related to pre-hospital advanced airway management not being performed.

**Results:**

We registered data from 1081 cases in which the pre-hospital critical care anaesthesiologists’ considered performing pre-hospital advanced airway management. The anaesthesiologists decided to withhold pre-hospital advanced airway management in 32.1% of these cases (n = 347). In 75.1% of these cases (n = 257) pre-hospital advanced airway management were withheld because of the patient’s condition and in 30.8% (n = 107) because of patient co-morbidity. The most frequently used alternative treatment was bag-mask ventilation, used in 82.7% of the cases (n = 287). Immediate complications related to the decision of not performing pre-hospital advanced airway management occurred in 0.6% of the cases (n = 2).

**Conclusion:**

We have illustrated the complexity of the critical decision-making associated with pre-hospital advanced airway management. This study is the first to identify the most common reasons why pre-hospital critical care anaesthesiologists sometimes choose to abstain from pre-hospital advanced airway management as well as the alternative treatment methods used.

## Introduction

### Background

Pre-hospital Advanced Airway Management (PHAAM) continues to be one of the main controversies in pre-hospital critical care and an international group of experts have selected the topic as one of the top five research priorities in pre-hospital care [1]. Sollid et al. have proposed a standardised way of registering and reporting data from PHAAM [2]. Until now, the focus of PHAAM-related research has largely been either on intubation success rates in different settings and different Emergency Medical Services (EMS) or on the potential effect, PHAAM may have on patient outcome [3-13]. Little attention has been paid to the critical decision making process involved in PHAAM. These decisions can be challenging even to pre-hospital critical care physicians [14,15]. Both we [9] and other authors [6,16] have reported pre-hospital critical care physicians’ reasons for performing PHAAM. Possible reasons for deciding against PHAAM may include anticipated difficult airway management, anticipated post-PHAAM complications, patient co-morbidity, lack of proper equipment, lack of sufficient training, the inability to get assistance and difficult operative environment (e.g. entrapped patient). To our knowledge, no authors have investigated this topic.

When choosing not to perform PHAAM, the pre-hospital critical care provider must have a viable alternative treatment option. We have identified no studies investigating the alternative airway management strategies used to treat these patients.

The incidences of complications related to PHAAM are not neglectable even in physician-staffed systems [9,11,17,18] and it is important that the pre-hospital care provider can identify the patients most likely to benefit from PHAAM. We have recently reported the incidences of different PHAAM-related complications in our anaesthesiologist-staffed pre-hospital critical care system [9] but the incidences of complications related to not performing PHAAM in a physician-staffed pre-hospital critical care system are unknown.

### Objectives

The main objective was to study the critical decision making process associated with the decision not to perform PHAAM. We did this by investigating:

1) the pre-hospital critical care anaesthesiologists’ reasons for considering performing PHAAM in the current population

2) the pre-hospital critical care anaesthesiologists’ reasons for not performing PHAAM

3) the methods used to treat the patients when PHAAM was not performed

4) the incidence of complications related to not performing PHAAM

5) the incidence of emergency department endotracheal intubation and difficult emergency department endotracheal intubation in this group of patients.

## Materials and methods

### Study design

We performed a prospective observational study in which we collected PHAAM-related data from physician-staffed pre-hospital critical care teams according to the consensus-based Utstein-style template proposed by Sollid et al. [2]. We have recently presented data from the cases where the pre-hospital critical care teams did perform PHAAM [9].

### Setting

The Central Denmark Region covers a mixed urban and rural area of approximately 13000 km^2^ with a population of 1.270.000, and an overall population density of 97.7 inhabitants/km^2^.

The EMS in the region is a two-tiered system based on 64 road ambulances staffed by emergency medical technicians (EMTs) supported by ten pre-hospital critical care teams staffed with an anaesthesiologist and a specially trained EMT. Rapid response vehicles deploy nine of the pre-hospital critical care teams; the tenth team staffs a HEMS helicopter. The pre-hospital critical care teams covered by this study employ approximately 90 anaesthesiologists as part-time pre-hospital physicians. There are no full-time pre-hospital critical care physicians in the region – all physicians primarily work in one of the five regional emergency hospitals or at the university hospital. Intensive care medicine is part of the Danish anaesthesiological curriculum. We have described the EMS system in our region in more detail elsewhere [9,19].

We collected data between February 1^st^ 2011 and November 1^st^ 2012.

### Participants

Inclusion criteria: Consecutive patients of all ages in whom the pre-hospital critical care anaesthesiologists considered performing PHAAM but decided against such interventions. Sollid et al. [2] define advanced airway management as any airway management beyond opening of the airway and the use of an oro-pharyngeal (*“Guedel*”) airway. We considered bag-mask-ventilation without the use of other adjuncts than an oro-pharyngeal airway not to be advanced airway management. In concordance with Sollid et al [2] we defined the use of a nasopharyngeal airway as advanced airway management.

Exclusion criteria: Patients in cardiac arrest where no treatment were initiated. Inter-hospital transfers.

### Endpoints and variables

Primary endpoints were 1) the pre-hospital critical care anesthesiologists’ reasons for considering PHAAM 2) the pre-hospital critical care anesthesiologists’ reasons for not performing PHAAM 3) methods used to treat the patient when withholding PHAAM 4) complications related to not performing PHAAM.

Secondary endpoints were 1) Emergency department endotracheal intubation (ED-ETI) 2) difficult ED-ETI.

We collected all core data proposed in the consensus-based template by Sollid et al. [2] and the variables were defined as in this template. Of special interest are the following definitions:

The indications for performing PHAAM as categorised by Sollid et al. [2] are: (1) decreased level of consciousness (2) hypoxemia (3) ineffective ventilation (4) existing airway obstruction (5) impending airway obstruction (6) combative or uncooperative patient (7) relief of pain or distress (8) cardio-pulmonary arrest (9) other.

For this study, we categorised the reasons for choosing not to perform PHAAM as

1. expected difficult airway management (as defined by the individual pre-hospital critical care anaesthesiologist)

2. difficult or limited access to the patient

3. short transport distance to the emergency department (as defined by the individual pre-hospital critical care anaesthesiologist)

4. aspects of the patient’s current medical condition

5. the patient’s co-morbidity

6. physician’s lack of training or experience with the type of patient in question

7. lack of proper equipment

8. no assistance available

9. other.

Reason number 4 includes both patients so critically ill or injured that advanced critical care is considered futile and patients so clinically unstable that the attending pre-hospital critical care anaesthesiologist assessed the risks associated with pre-hospital PHAAM to outweigh the potential benefits of a secure airway and controlled ventilation. Number 5 includes patients where co-morbidity renders PHAAM unethical (for instance patients with terminal chronic obstructive pulmonary disease (COPD) or terminal cancer) and where palliative care are more appropriate.

We analysed the reasons for not performing PHAAM in the following groups of non-cardiac arrest patients: a) trauma b) subarachnoid haemorrhage/stroke c) asthma/COPD and c) cardiac disease. We also analysed the reasons for not performing PHAAM in cardiac arrest patients.

The following alternatives to PHAAM were available:

1. spontaneous ventilation (with or without supplementary oxygen) *without* the use of airway manoeuvres (chin lift or jaw trust) or an oro-pharyngeal airway

2. spontaneous ventilation (with or without supplementary oxygen) *with* the use of airway manoeuvres

3. spontaneous ventilation (with or without supplementary oxygen) *with* the use of an oro-pharyngeal airway

4. bag-mask ventilation (BMV) *without* the use of an oro-pharyngeal airway

5. BMV *with* the use of an oro-pharyngeal airway.

Possible complications include (as defined in the template [2]): vomiting, aspiration of gastric content or blood to the lungs, hypoxia (oxygen saturation < 90%), hypotension (systolic blood pressure < 90 mmHg) or bradycardia (pulse <60 beats per minutes).

The pre-hospital critical care teams measured oxygen saturation, heart rate and blood pressure by using a LifePak 12 monitor (*Physio-Control, Redmond, USA*).

We gathered information about ED-ETI by linking pre-hospital data to the electronic patient journal systems in the region. The patients were categorised as 1a) ED-ETI performed – easy, 1b) ED-ETI performed – difficult, 2) ED-ETI not performed. We defined difficult endotracheal intubation according to the latest version of the *“Practice guidelines for management of the difficult airway”* by the American Society of Anesthesiologists [20] as more than one attempt needed to successfully perform endotracheal intubation.

### Data sources and data collection

We collected data from eight pre-hospital critical care teams, including the HEMS. The anaesthesiologists in the participating teams filled in a registration form containing all the core data recommended by Sollid et al. [2] as well as the study-specific variables listed above. A translated version of the registration form is available as Additional file 1. We have previously described the data collection process in more detail [9].

### Bias

To reduce the risk of recall bias and selection bias, the primary investigator reviewed the registration forms on a day-to-day basis looking for missing forms or incomplete data sets. We crosschecked the registration forms with the standard pre-hospital records from the pre-hospital critical care teams to ensure the highest possible data coverage. In cases of missing data or inconsistencies, we asked the attending pre-hospital critical care anaesthesiologists to provide additional details for clarification.

### Statistical methods

We analysed the data in the statistical program *Stata12 (StataCorpLP).* Because of the rigorous crosschecking and day-to-day control, missing data were rare. If the missing data were unobtainable, we performed complete case analyses.

### Ethics

No patients had their treatment altered because of the study. All anaesthesiologists participated in the study on a voluntary basis – there were no refusals. The study did not involve any alterations from normal practice and according to Danish law, it did not need the approval of the Regional Ethics Committee, nor did we need the patients’ consent to register and publish the data. The Danish Data Protection Agency approved the study (Journal number 2013-41-1462).

## Results

### Participants

**Figure 1:**
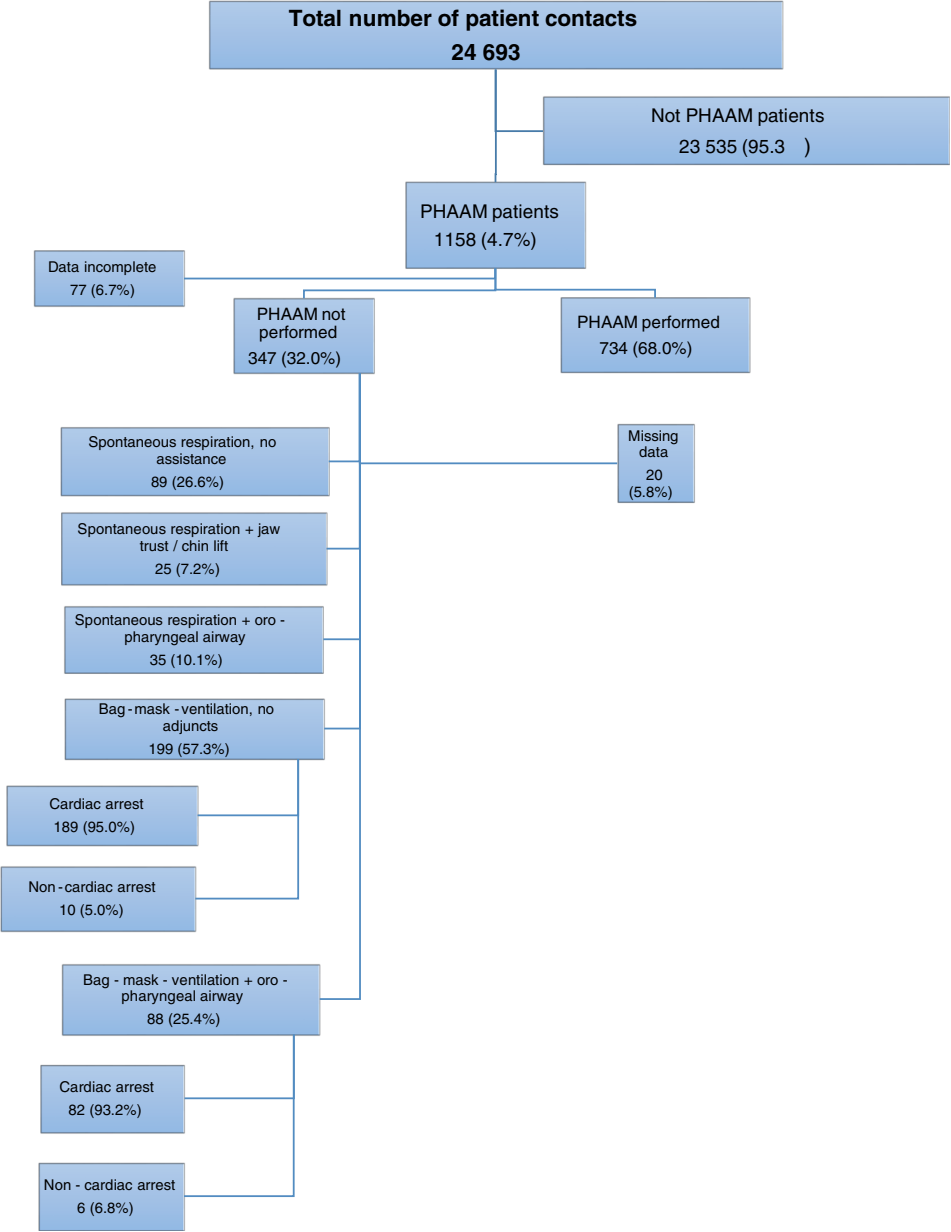
**Patient flow.** Numbers (%). PHAAM: Pre-hospital Advanced Airway Management. RSI: Rapid Sequence Intubation.

Figure 1 is a flow diagram showing that during the 21 months, the participating pre-hospital critical care teams treated 24 693 patients. The teams registered data from 1081 possible PHAAM cases corresponding to 93.3% of the possible PHAAM patients. The physicians decided to withhold PHAAM in 32.1% of these cases (n = 347).

### Descriptive data

We display the demographics, co-morbidity data and patient characteristics of the 347 patients in whom the pre-hospital critical care physicians decided against PHAAM as Additional file 2. The patients mean age was 61.4 years (range 0-97), 3.5% (n = 12) were under the age of 16 and 0.9% (n = 3) were under the age of 2.

### Pre-hospital critical care anesthesiologists’ reasons for considering PHAAM

We present pre-hospital critical care anesthesiologists’ reasons for considering PHAAM in Table 1.

**Table 1:** Pre-hospital critical care anaesthesiologists’ reasons for considering pre-hospital advanced airway management (n = 347)

**Indication**	Total*	**%**
Decreased level of consciousness	122	35.2
Hypoxemia	67	19.3
Ineffective ventilation	55	15.9
Existing airway obstruction	4	1.2
Impending airway obstruction	27	7.8
Anaesthesia to combative or agitated patient	3	0.9
Anaesthesia for pain relief or distress	5	1.4
Cardiac arrest	197	56.8
Other indications	10	2.9

*Physicians may have more than one reason for considering pre-hospital advanced airway management.

### Pre-hospital critical care anesthesiologists’ reasons for not performing PHAAM

Table 2 displays the anaesthesiologists’ reasons for withholding PHAAM. Please note that several reasons may apply to each patient. PHAAM were withheld *solely* because of a short distance to the ED in 6.6% (n = 23) of these patients. Of these, 87.0% (n = 20) were endotracheally intubated in the ED.

**Table 2:** Prehospital critical care anaesthesiologists’ reasons for pre-hospital critical care anaesthesiologists postponing pre-hospital advanced airway management (PHAAM) (n = 347)

Reason for postponing/withholding PHAAM*	**Patients in total**	ETI** in ED***	**Difficult ETI in ED**
	**Number (% of the 347 patients)**	**Number (% of total number in row)**	
			**Number (% of total number in row)**
Expected difficult PHETI****	19 (5.5)	10 (52.6)	2 (20.0)
Difficult access to patient	4 (1.2)	1 (25.0)	0
Short transport time to the ED	64 (18.4)	30 (46.9)	3 (10.0)
The patient’s condition	257 (75.1)	15 (5.8)	1 (6.7)
Patient co-morbidity	107 (30.8)	3 (2.8)	1 (33.3)
Insufficient PHAAM training	0	0	0
Insufficient equipment available	1 (0.3)	1 (100.0)	1 (100%)
No assistance available	4 (1.2)	2 (50.0)	0
Other	0	0	0

*Patients may fall into more than one category.

**Endotracheal Intubation.

***Emergency Department.

****Pre-hospital Endotracheal Intubation.

Additional file 3 shows details of the anaesthesiologists’ reasons for withholding PHAAM in different patient categories as well as the different groups of patients according to the physicians’ reasons for not performing PHAAM.

### Methods used to treat the patients when not performing PHAAM

Figure 1 shows the overall incidences of different alternative treatment modalities. In total, 94.4% (n = 271) of the 287 patients managed with bag-mask ventilations were in cardiac arrest (Figure 1). We present the methods used according to patient subgroups and according to physicians’ reasons for not performing PHAAM in Additional file 3.

### Complications related to not performing PHAAM

Two (0.6%) of the 347 patients not treated with PHAAM suffered aspiration of gastric content or blood into the lungs. We recorded no other immediate airway complications or complications related to the decision not to perform PHAAM.

### Endotracheal intubation in the Emergency Department

We display the incidence of ED-ETI according to physicians’ reasons for not performing PHAAM in Table 2. We did not register the incidences of complications associated with ED-ETI.

### The incidence of difficult ED-ETI

We display the incidence of difficult ED-ETI according to physicians’ reasons for not performing PHAAM in Table 2.

## Discussion

### Pre-hospital critical care anesthesiologists’ reasons for not performing PHAAM

The most common reason for withholding PHAAM in our system is patient condition and patient co-morbidity. Unfortunately, our data do not allow us to distinguish between cases where PHAAM were not necessary, cases where PHAAM were considered associated with too high a risks of complications and cases where the attending anaesthesiologist deemed PHAAM futile or unethical. Nevertheless, the current study provides the first important knowledge into why pre-hospital critical care anaesthesiologists sometime withhold PHAAM.

Our current results show that a high percentage of potential PHAAM-patients not treated with PHAAM were in cardiac arrest. It is important to stress that the decision not to perform PHAAM is not the same as deciding not to perform cardio-pulmonary resuscitation (CPR). Ventilating the patient with BMV during CPR may be in accordance with current guidelines [21]. These guidelines stress the importance of delivering chest compressions with as few and as short interruptions as possible. Not attempting PHAAM when BMV is sufficient, might therefore be a reasonable choice. It may also be a sensible decision to delay PHAAM until the attending pre-hospital anaesthesiologists, who is also team leader, has made sure that CPR is being performed correctly and that more pressing interventions are being carried out first. However, waiving or postponing PHAAM during CPR may also have drawbacks: 1) Not performing PHAAM excludes the possibility of providing continuous chest compressions. This may result in increased hands-off-time during CPR 2) Current guidelines emphasise the value of having continuous end-tidal CO_2_ measurement during CPR to monitor the quality of CPR and to detect return of spontaneous circulation (ROSC) at an early stage [21]. This requires PHAAM (either PHETI or the installation of a SAD) to be performed. 3) Delaying PHAAM until the patient has achieved ROSC as the current guidelines also suggest, usually requires RSI, which may result in post-RSI hypotension or hypoxia [9]. But then again, some patients wake up following ROSC and may not need PHAAM. We do not know the optimal timing of PHAAM during or after CPR and it is likely to vary considerably from patient to patient. This adds to the complexity of these critical decisions.

It is noteworthy, that limited access to the patient is a rare reason for withholding PHAAM in our system. In the few case where this played a role in the decision-making, it was typically a cardiac arrest patient lying in a narrow place where PHAAM would be difficult but where BMV was possible.

In a small but significant proportion of our patients, PHAAM where not performed because of a short transport distance from the scene to the ED. This might be a sensible solution, especially if there is an increased risk of difficult PHETI or post-RSI complications, or if the pre-hospital critical care anaesthesiologists is occupied performing even more urgent interventions on the patient. On the other hand, even a few minutes transfer from the scene to the ED may prove disastrous if the patient has a severe airway or respiratory problem. Furthermore, even though transfer time itself may be short, patient packaging and loading on scene and patient handover in the ED may add to the ETI delay.

Our 1.8% incidence of PHAAM not performed because the attending pre-hospital critical care anaesthesiologist anticipated a difficult-to-manage airway is in contrast to the approximately 22% incidence of intubations requiring more than one intubation attempt, previously reported from our system [9]. We have argued the need for an increased first pass intubation success rate in our system [9]. The current results indicates that one possible way to achieve this may be by implementing a pre-PHAAM check-list to remind the pre-hospital critical care team of the importance of optimal pre-PHAAM preparations (including formal airway evaluations) and first pass success.

### Methods used to treat the patients when not performing PHAAM

The fact that the most common method of treating patients in cases where the pre-hospital critical care physician withheld PHAAM were BMV corresponds to the high incidence of cardiac arrest in our material.

### Complications related to not performing PHAAM

The risks associated with PHETI, which in our system is comparable to those associated with RSI in British and North-American EDs [9], must be balanced against the risks of aspiration, hypoventilation and hypoxia if PHETI is not performed. The incidence of immediate airway-related complications in the cases where the pre-hospital critical care anaesthesiologists did not performed PHAAM seems to be very low in our system. It is important to stress however, that due to the nature of this study these results cannot be compared with the incidences of complications previously found in the group where PHAAM was performed [9]. It is essential to note, that the patients in this study were highly selected; experienced pre-hospital critical care anaesthesiologists chose not to perform PHAAM because they judged it to be in the patients’ best interest. Never the less, our results indicate that the pre-hospital critical care anaesthesiologists in this study seldom experience complications following their decisions not to perform PHAAM. One additional explanation for this may be that when they do experience complications, they solve the problem by performing PHAAM. We do not know the incidence of postponing PHAAM in these cases nor the consequences of these decisions. Based on the pre-hospital critical care anaesthesiologists’ journal entries it appears to be a rare event.

### Endotracheal intubation in the Emergency Department

It is interesting, that the in-hospital anaesthesiologists in the ED performed ETI in almost 90% of the patients in whom the only reason for not performing PHETI was a short transport distance to the ED. Our data do not allow us to calculate the time delay to ETI in those patients who had their trachea intubated in the ED. We would like to stress that we do not believe that a “maximum acceptable delay” can be defined in these situations. In our opinion, postponing PHAAM solely because of a short distance to the ED is a debateable practice.

It is also worth noticing that only one fifth of the patients assessed by the pre-hospital critical care physician as having a potential difficult airway proved to have a difficult ED-ETI. These results supports our previous notion that our system may need additional critical decision-making aids to further improve system performance and patient safety related to PHAAM.

### Limitations

The main limitation of the current study is that the attending physicians registered the data. They are therefore subject to registration bias (systematic errors in the registration of data) or recall bias. The high capture rate reduces the risk of selection bias. We tried to reduce the effects of this further as described in the Bias section.

We did not introduce a set of criteria for when the attending anaesthesiologists ought to consider performing PHAAM. We wanted to describe our services as they currently are; with no overall guidelines or SOPs for PHAAM. This may very well have influenced the data collected, as the reasons for considering performing PHAAM are more than likely to vary between the pre-hospital critical care anaesthesiologists. However, we feel that this depicts the current situation in our system.

As with the other parts of this prospective study [9], we did not design it to detect possible mid- or long-term complications related to not performing PHAAM (i.e. pneumonia or acute respiratory distress syndrome). This may have led us to underestimate the true overall incidence of complications. On the other hand, such follow-up studies may overestimate the incidence of complications, as it would be impossible to distinguish between complications that were a result of not performing PHAAM and those who were a result of events that happened prior to the decision not to perform PHAAM.

Missing data are rare except for the 25% of missing reasons for not performing PHAAM in cardiac arrest cases. We do not know why missing data are more common in this group, but based on the review of the registration forms and the patients’ pre-hospital charts the by far most common reason for not performing PHAAM in this group of patients were the patient’s condition (i.e. the CPR were terminated before the attending anaesthesiologist considered PHAAM to be necessary).

It is vitally important to understand that this study does not answer the question whether PHAAM and PHETI should be performed or not. The low incidence of complications associated with not performing PHAAM should not be generalised to the whole populations of pre-hospital critical care patients. Caution is therefor advised when interpreting our results.

### Generalisability

This was part of a larger study from one homogenous Danish system of anaesthesiologist-staffed pre-hospital critical care teams. This limits the ability to generalise the findings to other systems with different staffing, caseload or case mix. Nevertheless, we believe that our results may be of use to other both physician- and paramedic-staffed pre-hospital services. We also believe that they may be useful when trying to improve patient safety in different pre-hospital systems.

### Perspectives

Further studies are required to gain more knowledge into the critical decision making process involved in PHAAM and especially to establish whether introducing SOPs and checklists in physician-staffed pre-hospital systems can reduce the incidence of complications and ultimately improve patient safety and outcome [22]. One may speculate that our system based on experienced anaesthesiologists working as pre-hospital critical care physicians would benefit from the implementation of SOPs and checklists for PHAAM. We wonder however, if not PHAAM performed by experienced anaesthesiologists who are able to tailor the treatment to each individual patient based on the patient’s clinical condition, co-morbidity and risk profile may have its advantages compared to strictly SOP-based care. This may especially apply when it comes to choosing which patients to treat with PHAAM, how to perform PHAAM and how to avoid PHAAM-related complications. In the end, the decision of how to achieve the maximum level of PHAAM-related patient safety in an EMS/HEMS is likely to depend on both the patient population served by the different systems and on the level of PHAAM expertise available in different systems.

## Conclusions

We have illustrated some of the complexity of the critical decision making process associated with pre-hospital advanced airway management. We have also identified the reasons for not performing pre-hospital advanced airway management and the alternative treatment methods used in our system. Immediate complications related to the decision of not performing pre-hospital advanced airway management were rare.

## Abbreviations

PHAAM: Pre-hospital advanced airway management; EMS: Emergency medical service; HEMS: Helicopter emergency medical service; ETI: Endotracheal intubation; PHETI: Pre-hospital endotracheal intubation; EMT: Emergency medical technicians; RSI: Rapid sequence intubation; ED: Emergency department; GCS: Glasgow coma scale.

## Competing interests

The authors declare that they have no competing interests.

## Authors’ contributions

LR and TMH conceived the study. LR, TMH and ET designed the study. LR performed the data collection and –management. All authors contributed to data analysis and -interpretation. LR drafted the manuscript. TMH, HK and ET revised the manuscript critically for important intellectual content. All authors read and approved the final version of the manuscript for publication.

## Authors’ information

LR is a research fellow at the Norwegian Air Ambulance Foundation, consultant anaesthesiologist at the Viborg Regional Hospital and a pre-hospital critical care physician in the Central Denmark Region. He currently holds the post as Programme Director for the *Scandinavian Society of Anaesthesiology and Intensive Care Medicine Advanced Educational Programme in Emergency Critical Care*.

TMH is a consultant anaesthesiologist at the Aarhus University Hospital, Lead Clinician of the Pre-hospital Critical Care Team in Aarhus and a pre-hospital critical care physician in the Central Denmark Region.

HK is a consultant anaesthesiologist and professor of emergency medicine at the Aarhus University Hospital.

ET is a consultant anaesthesiologist and professor of anaesthesiology at the Aarhus University Hospital.

## Supplementary Material

Additional file 1 Registration form. Click here for file

Additional file 2 Demographic data and patients' characteristics. Click here for file

Additional file 3 Further patients' and treatment details. Click here for file
