# Supplementary material for: Refraining from pre-hospital advanced airway management: a prospective observational study of critical decision making in an anaesthesiologist-staffed pre-hospital critical care service
Source: Scand J Trauma Resusc Emerg Med. 2013 Oct 25;21:75. doi: 10.1186/1757-7241-21-75 (PMC4176298; doi:10.1186/1757-7241-21-75)
Supplement: Additional file 2 — Demographic data and patients' characteristics. [file 1757-7241-21-75-S2.pdf]

Additional file 2

Rognås et al., "Refraining from pre-hospital advanced airway management: a prospective observational study from an anaesthesiologist-staffed pre-hospital critical care service."

Demographic data

	Numbers	%
Total number of patients included (N)	347	
Males	211	60.8
ASA-PS*-score mean	2.20	1-4 (range)
Pre-existing cardiac disease	75	21.7
Pre-existing hypertension	22	6.4
Pre-existing COPD**	51	14.7
Pre-existing diabetes	21	6.1
Pre-existing neurological disease	44	12.7
Other pre-existing disease	99	28.6

*American Society of Anesthesiologists Physical Status
** Chronic Obstructive Pulmonary Disease.

Patients characteristics (N=347)

Patient category	Number	%
Isolated traumatic brain injury	5	1.4
Multitrauma (blunt)	4	1.2
Strangulation /suffocation	3	0.9
Burns	2	0.6
Other blunt trauma	2	0.6
Blunt trauma, total	16	4.5
Penetrating trauma	1	0.3
Trauma, total	17	4.9
Cardiac arrest	203	58.5
Cardiac (excluding cardiac arrest)	21	6.1
Asthma / COPD*	25	7.2
Stroke / subarachnoid hemorrhage	34	9.8
Oto- rhino- laryngology	1	0.3
Other patient categories	74	21.3
First respiratory rate below 8	198	57.1
First respiratory rate over 30	19	5.5
First oxygen saturation below 90%	325 **	93.6
First heart rate below 40	190	54.8
First heart rate over 180	3	0.9
First systolic blood pressure below 90 mmHg	200	57.6
First GCS*** 3-8	274	80.0
First GCS 9-13	23	6.6
First GCS 14-15	43****	12.4

*Chronic Obstructive Pulmonary Disease
**115 with supplementary oxygen
***Glasgow Coma Scale score
**** GCS data is missing on 7 patients
