# Supplementary material for: Refraining from pre-hospital advanced airway management: a prospective observational study of critical decision making in an anaesthesiologist-staffed pre-hospital critical care service
Source: Scand J Trauma Resusc Emerg Med. 2013 Oct 25;21:75. doi: 10.1186/1757-7241-21-75 (PMC4176298; doi:10.1186/1757-7241-21-75)
Supplement: Additional file 3 — Further patients' and treatment details. [file 1757-7241-21-75-S3.pdf]

Additional file 3

Rognås et al., "Refraining from pre-hospital advanced airway management: a prospective observational study from an anaesthesiologist-staffed pre-hospital critical care service."

Non-cardiac arrest patients with an initial GSC<9 where pre-hospital advanced airway management (PHAAM) where not performed

**Patient category**	**PHAAM not performed** Number(%)	**Physicians' reasons for not performing PHAAM** Number*		**Alternative airway management method used** Number	
Trauma (n=134)	9 (6.7)	Patients condition<br>Short transport time to ED**<br>Expected difficult PHETI***	8<br>2<br>1	Spontaneous respiration, no assistance<br>Spontaneous respiration+ oropharyngeal airway<br>Bag-mask ventilation, no adjuncts<br>Bag-mask ventilation+oropharyngeal airway	2<br>1<br>4<br>2
SAH**** / stroke (n=70)	28 (40.0)	Patients condition<br>Co-morbidity<br>Short transport time to ED<br>Expected difficult PHETI<br>Difficult access to patient	13<br>15<br>10<br>3<br>1	Spontaneous respiration, no assistance<br>Spontaneous respiration+ jaw trust /chin lift<br>Spontaneous respiration+ oropharyng. airway<br>Bag-mask ventilation, no adjuncts<br>Bag-mask ventilation+ oropharyngeal airway	12<br>5<br>5<br>3<br>3
Asthma / COPD***** (n=30)	9 (30.0)	Patients condition<br>Co-morbidity	4<br>6	Spontaneous respiration, no assistance<br>Bag-mask ventilation, no adjuncts<br>Bag-mask ventilation+ oropharyngeal airway	2<br>4<br>3
Cardiology (n=8)	4 (50.0)	Patients condition<br>Short transport time to ED<br>Expected difficult PHETI	3<br>1<br>1	Spontaneous respiration, no assistance<br>Spontaneous respiration+ jaw trust /chin lift<br>Bag-mask ventilation, no adjuncts	1<br>1<br>2

* More than one reason may apply to each patient
** Emergency Department
***Pre-hospital Endotracheal intubation
****Subarachnoid Haemorrhage
*****Chronic Obstructive Pulmonary Disease

Pre-hospital critical care anaesthesiologists' reasons for not performing pre-hospital advanced airway management on cardiac arrest patients (n=271).

**Reason for not performing PHAAM***	**Number****	**%**
Patients condition	157	57.9
Co-morbidity	57	21.0
Short transport time to ED***	16	5.9
Expected difficult PHETI ****	4	1.5
Difficult access to patient	2	0.7
Lack of training	0	
Lack of equipment	0	
Lack of assistance	0	
Data missing	68	25.1

*Pre-hospital advanced airway management
** More than one reason may apply to each patient
*** Emergency Department
**** Pre-hospital Endotracheal Intubation

Additional file 3

Rognås et al., "Refraining from pre-hospital advanced airway management: a prospective observational study from an anaesthesiologist-staffed pre-hospital critical care service."

Patient categories when pre-hospital advanced airway management (PHAAM) were withheld

Reason for withholding PHAAM	Patient category*	Numbers (%)
**The patient's condition** (n=257)	Trauma	11 (4.3)
	Cardiac arrest (CA)	157 (61.1)
	Cardiological disease (not CA)	24 (9.3)
	Asthma / Chronic Obstructive Pulmonary Disease	13 (5.1)
	Oto-rhino-laryngological disease	0
	Subarachnoid haemorrhage / stroke	16 (6.2)
	Other	51 (19.8)
**Patient comorbidity** (n=107)	Trauma	2 (1.9)
	Cardiac arrest (CA)	57 (53.3)
	Cardiological disease (not CA)	5 (4.7)
	Asthma / Chronic Obstructive Pulmonary Disease	18 (16.8)
	Oto-rhino-laryngological disease	0
	Subarachnoid haemorrhage / stroke	18 (16.2)
	Other	16 (15.0)
**Short transport time to ED**** (n=64)	Trauma	8 (12.5)
	Cardiac arrest (CA)	16 (25.0)
	Cardiological disease (not CA)	4 (6.3)
	Asthma / Chronic Obstructive Pulmonary Disease	7 (10.9)
	Oto-rhino-laryngological disease	1 (1.6)
	Subarachnoid haemorrhage / stroke	13 (20.3)
	Other	24 (37.5)
**Expected difficult PHETI***** (n=19)	Trauma	5 (26.3)
	Cardiac arrest (CA)	4 (21.1)
	Cardiological disease (not CA)	3 (15.8)
	Asthma / Chronic Obstructive Pulmonary Disease	1 (5.3)
	Oto-rhino-laryngological disease	1(5.3)
	Subarachnoid haemorrhage / stroke	3(15.8)
	Other	6 (31.6)
**Difficult access to patient** (n=4)	Trauma	0
	Cardiac arrest (CA)	2 (50.0)
	Cardiological disease (not CA)	0
	Asthma / Chronic Obstructive Pulmonary Disease	1 (25.0)
	Oto-rhino-laryngological disease	0
	Subarachnoid haemorrhage / stroke	1 (25.0)
	Other	1 (25.0)
**No assistance available** (n=4)	Trauma	1 (25.0)
	Cardiac arrest (CA)	0
	Cardiological disease (not CA)	1 (25.0)
	Asthma / Chronic Obstructive Pulmonary Disease	0
	Oto-rhino-laryngological disease	1 (25.0)
	Subarachnoid haemorrhage / stroke	1 (25.0)
	Other	0
**Insufficient equipment** (n=1)	Trauma	0
	Cardiac arrest (CA)	0
	Cardiological disease (not CA)	0
	Asthma / Chronic Obstructive Pulmonary Disease	0
	Oto-rhino-laryngological disease	1 (100.0)
	Subarachnoid haemorrhage / stroke	0
	Other	0

*Patients may fall into more than one category
** Emergency Department
***Pre-hospital Endotracheal Intubation

Additional file 3

Rognås et al., "Refraining from pre-hospital advanced airway management: a prospective observational study from an anaesthesiologist-staffed pre-hospital critical care service."

Alternative methods used to treat the patient when pre-hospital advanced airway management (PHAAM) were withheld or postponed

Reason for withholding PHAAM*	Alternative methods used	Numbers (%)
**The patient's condition** (n=257)	Spontaneous respiration, no assistance<br>Spontaneous respiration with jaw trust /chin lift<br>Spontaneous respiration with oropharyngeal airway<br>Bag-valve-mask ventilation, no adjuncts<br>Bag-valve-mask ventilation with oropharyngeal airway<br>Missing data	47 (18.3)<br>6 (2.3)<br>16 (6.2)<br>124 (48.2)<br>50 (19.5)<br>14 (5.4)
**Patient comorbidity** (n=107)	Spontaneous respiration, no assistance<br>Spontaneous respiration with jaw trust /chin lift<br>Spontaneous respiration with oropharyngeal airway<br>Bag-valve-mask ventilation, no adjuncts<br>Bag-valve-mask ventilation with oropharyngeal airway<br>Missing data	21 (19.6)<br>5 (4.7)<br>11 (10.3)<br>46 (43.0)<br>22 (20.6)<br>2 (1.9)
**Short transport time to ED**** (n=64)	Spontaneous respiration, no assistance<br>Spontaneous respiration with jaw trust /chin lift<br>Spontaneous respiration with oropharyngeal airway<br>Bag-valve-mask ventilation, no adjuncts<br>Bag-valve-mask ventilation with oropharyngeal airway<br>Missing data	15 (23.4)<br>10 (15.6)<br>6 (9.4)<br>22 (34.4)<br>10 ((15.6)<br>1 (1.6)
**Expected difficult PHETI***** (n=19)	Spontaneous respiration, no assistance<br>Spontaneous respiration with jaw trust /chin lift<br>Spontaneous respiration with oropharyngeal airway<br>Bag-valve-mask ventilation, no adjuncts<br>Bag-valve-mask ventilation with oropharyngeal airway<br>Missing data	4 (21.1)<br>2 (10.5)<br>2 (10.5)<br>6 (31.6)<br>3 (15.8)<br>2 (10.5)
**Difficult access to patient** (n=4)	Spontaneous respiration, no assistance<br>Spontaneous respiration with jaw trust /chin lift<br>Spontaneous respiration with oropharyngeal airway<br>Bag-valve-mask ventilation, no adjuncts<br>Bag-valve-mask ventilation with oropharyngeal airway<br>Missing data	1 (25.0)<br>0<br>0<br>1 (25.0)<br>2 (50.0)<br>0
**No assistance available** (n=4)	Spontaneous respiration, no assistance<br>Spontaneous respiration with jaw trust /chin lift<br>Spontaneous respiration with oropharyngeal airway<br>Bag-valve-mask ventilation, no adjuncts<br>Bag-valve-mask ventilation with oropharyngeal airway<br>Missing data	0<br>2 (50.0)<br>0<br>0<br>1 (25.0)<br>1 (25.0)
**Insufficient equipment** (n=1)	Spontaneous respiration, no assistance<br>Spontaneous respiration with jaw trust /chin lift<br>Spontaneous respiration with oropharyngeal airway<br>Bag-valve-mask ventilation, no adjuncts<br>Bag-valve-mask ventilation with oropharyngeal airway<br>Missing data	1 (100.0)<br>0<br>0<br>0<br>0<br>0
**In total** (n=456)	Spontaneous respiration, no assistance<br>Spontaneous respiration with jaw trust /chin lift<br>Spontaneous respiration with oropharyngeal airway<br>Bag-valve-mask ventilation, no adjuncts<br>Bag-valve-mask ventilation with oropharyngeal airway<br>Missing data	89 (26.6)<br>25 (7.2)<br>35 (10.1)<br>199 (57.3)<br>88 (25.4)<br>20 (5.8)

*Patients may fall into more than one category
** Emergency Department
***Pre-hospital Endotracheal Intubation
